# Automatic classification of literature in systematic reviews on food safety using machine learning

**DOI:** 10.1016/j.crfs.2021.12.010

**Published:** 2021-12-26

**Authors:** Leonieke M. van den Bulk, Yamine Bouzembrak, Anand Gavai, Ningjing Liu, Lukas J. van den Heuvel, Hans J.P. Marvin

**Affiliations:** Wageningen Food Safety Research, Akkermaalsbos 2, 6708, WB, Wageningen, the Netherlands

**Keywords:** Literature reviews, Text mining, Classification models, Document screening, Artificial intelligence, Food safety hazards

## Abstract

Systematic reviews are used to collect relevant literature to answer a research question in a way that is clear, thorough, unbiased and reproducible. They are implemented as a standard method in the domain of food safety to obtain a literature overview on the state-of-the-art research related to food safety topics of interest. A disadvantage to systematic reviews, however, is that this process is time-consuming and requires expert domain knowledge. The work reported here aims to reduce the time needed by an expert to screen all possible relevant articles by applying machine learning techniques to classify the articles automatically as either relevant or not relevant. Eight different machine learning algorithms and ensembles of all combinations of these algorithms were tested on two different systematic reviews on food safety (i.e. chemical hazards in cereals and leafy greens). The results showed that the best performance was obtained by an ensemble of naive Bayes and a support vector machine, resulting in an average decrease of 32.8% in the amount of articles the expert has to read and an average decrease in irrelevant articles of 57.8% while keeping 95% of the relevant articles. It was concluded that automatic classification of the literature in a systematic literature review can support experts in their task and save valuable time without compromising the quality of the review.

## 1 Introduction

A systematic review is an approach to collect a complete and exhaustive summary of current literature to answer a specific research question in a way that is clear, thorough, reproducible and unbiased (Higgins et al., 2019). Systematic reviews follow a fixed procedure. They entail gathering research using a priori defined criteria and describing and analyzing the reported results of the deemed relevant literature in a systematic way. This in contrast to the traditional narrative reviews where the process of literature selection and assessment criteria are often not explicit, which can lead to selection and performance bias (EFSA, 2010; Higgins and Green, 2011). These biases arise when there is no extensive, systematic way of searching for literature and a selective strategy of reporting results of relevant studies is often based on the interpretation of the reviewer. While expert judgment is still involved in conducting systematic review, the structured processes are designed to minimize bias and increase transparency with respect to expert judgements.

Both the European Food Safety Authority (EFSA) and the United States Department of Agriculture (USDA) adopted the use of systematic reviews as a standardized method to identify research on food and feed safety to ensure the selection of robust and relevant studies while increasing credibility and transparency (EFSA, 2010; Fungwe et al., 2009). Independent risk assessments of the food chain are performed and advice on existing and emerging food risks is given. The knowledge gained in the systematic reviews provide European and American authorities with input for prioritizing future monitoring activities due to new information and trends in consumption behavior or processing methods of food and feed.

A systematic review entails four main steps: (1) Formulating a research question and establishing a reproducible methodology for the review, (2) creating a search query to retrieve literature from databases that are applicable to the research question, (3) screening the collected literature for its relevance based on the titles and abstracts and (4) collecting and analyzing the results reported in the relevant literature. These steps can be very time-consuming, making a systematic review a costly undertaking. The third step alone already consists of reading through hundreds or even thousands of papers and assessing whether they are relevant for the case at hand. Since in the future more and more research will become available, the screening and assessment of the literature will become an increasingly bigger task.

To reduce human burden and resources required as well as increase the speed at which results are produced, machine learning algorithms are becoming an increasingly popular tool in a lot of areas. When it comes to text as input, a specialized part of machine learning called text mining has been on the rise (Gupta and Lehal, 2009; Talib et al., 2016; Hassani et al., 2020; Jung and Lee, 2020). Text mining refers to the process of automatically extracting information from text that is meaningful and nontrivial (Feldman and Sanger, 2007; Jo, 2019). It has already been successfully applied in many domains, for example in language translation (Wu et al., 2016; Aharoni et al., 2019; Popel et al., 2020), spam detection in emails (Dada et al., 2019; Zamir et al., 2020; Akinyelu, 2021), sentiment analysis and opinion mining (Ain et al., 2017; Yue et al., 2019; Liu, 2020) and automatic summarization (Aries et al., 2019; Zhang et al., 2020; El-Kassas et al., 2021).

Text mining can also be a valuable tool in systematic reviews by assisting the reviewers in the screening of the set of collected literature for its relevance. Just as the reviewers judge the literature based on their titles and abstracts, text mining can be used to automatically classify the literature as relevant or not relevant based on the combined text of the title and abstract as its input.

Over the past two decades multiple studies have explored the use of machine learning to classify the relevancy of literature for systematic reviews. One of the first was the work by Cohen et al. (2006) who explored if automatic classification of medical articles on efficacy of drugs could reduce time spent by the experts. It used the title and abstract together with the keywords and publication type to create bag-of-words feature vectors. The feature vectors were fed to an ensemble of one-layer neural networks (NN) for classification. They could reduce the amount of articles for the reviewer to screen with an average of 23% with a mean precision of 10% and a mean recall of 95%.^1^ Wallace et al. (2010) had the same goal of reducing time for the reviewer in mind for their research and applied an ensemble of support vector machines (SVM) on biomedical literature. They used the title, abstract and keywords in a term frequency–inverse document frequency (TF-IDF) feature vector as input for the model. With a recall of 1 they reduced the amount of articles to review by 46% on average. The work by Bekhuis and Demner-Fushman (2012) showed a comparison of a k-Nearest Neighbors (KNN) classifier, naive Bayes (NB) and SVM on the classification of medical systematic reviews. They concluded evolutionary SVMs worked best, using bag-of-words feature vectors with a recall of 95%, a precision of 11% and a reduction in articles to be screened of 46%. In 2014, García Adeva et al. (2014) tested four classifiers: NB, KNN, SVM and Rocchio. The data set again consisted of medical systematic reviews. It was concluded that the SVM worked best with a recall value of 70% and a precision of 72%, reducing the amount of articles that need screening with 77% at the cost of losing 30% of relevant articles. Timsina et al. (2016) retested four data sets used by Cohen et al. (2006) using three types of SVMs (linear, polynomial and evolutionary), NB and a single-layer NN. They tested their performance on two feature types, TF-IDF features and Unified Medical Language System (UMLS) features consisting of only those words occurring in medical vocabularies. The polynomial SVM performed best in all data sets with both features types, leading to an average reduction in articles of 59% with an average recall of 99% using the UMLS features.

The studies mentioned above have all been applied in the domain of medicine, mostly as systematic reviews play a very important role in evidence-based medicine (Sauerland and Seiler, 2005). In 2018, Jaspers et al. (2018) presented a report on the possible applications of machine learning in systematic reviews within EFSA. They evaluated the automation of screening abstracts by testing four different classifiers and all possible ensembles on the data of three systematic food safety reviews. The classifiers tested were an SVM, two-layer NN, random forest (RF) and gradient boosting (GB). Furthermore, they tested two different techniques of feature creation: Bag of words and topic modeling through latent Dirichlet allocation. They concluded that ensembles often performed best, but there was no optimal solution to the combination of models in the ensemble over the tested cases. RFs and NNs were the best individual classifiers and all classifiers had to use data augmentation to counteract the imbalance in the data in order to perform optimally. Using an RF and topic modeling they reduced the amount of literature to be screened by approximately 60% with an average recall of 80%.

The aim of this study was to further the research on automatic classification of scientific literature in the screening stage of systematic reviews, specifically in the domain of food safety. In contrast to the systematic reviews in medicine and the cases presented by EFSA, which in many instances contain thousands of articles, the amount of literature in food safety can often be significantly smaller. The amount of data can have a pronounced effect on the classifier performance. The efficacy of relevancy classification in those cases that only contain a few hundred articles was tested. Eight different algorithms ranging from classical text classification algorithms like an SVM to the current state-of-the-art on text classification like the BERT algorithm were implemented to cover a wide range of classifiers. The combination of the title and abstract of an article retrieved by a manually created search query within a specific topic of food safety was classified as either relevant or not relevant. The final goal of the research was to assist the experts and save valuable time, not to replace them entirely.

The data of two systematic reviews performed for the Netherlands Food and Consumer Product Safety Authority (NVWA) were used for this study: one on cereals (Kluche et al., 2020) and one on leafy greens (Banach et al., 2019). The goal of the reviews was the identification of chemical hazards in their respective supply chains. The systematic literature reviews were performed using search queries defined by experts applied to the databases of Scopus^2^ and Web of Science^3^ for the years 2008–2018 for the topic of cereals and 2009–2019 for the topic of leafy greens.

## 2 Materials and methods

### 2.1 Machine learning algorithms

Eight different machine learning algorithms were trained to classify the relevance of an article in a supervised way. The algorithms were selected based on the fact that they are suitable for binary classification, they can handle text data as input and that they are easily implemented through freely available coding packages. All algorithms were implemented in Python 3.7.^4^ All code is available on GitHub (see Appendix A). In the sections below each algorithm is explained in short. For more detailed explanations the reader is referred to the cited references.

#### 2.1.1 Logistic regression (LR)

LR is an algorithm that calculates the probability of an event by applying a log-odds function on the dependent variable (Menard, 2002; Peng et al., 2002; Hosmer et al., 2013). The log-odds function is the logarithm of the odds. Similar to linear regression, it is assumed that there exists a linear relationship between the independent variables of a data point, called features, and in this case the log-odds of the probability of the binary dependent variable, called the class:

(1) $$log\left(\frac{P\left(y_{i}=1|x_{i}\right)}{1-P\left(y_{i}=1|x_{i}\right)}\right)=\beta_{0}+\beta_{1}x_{i,1}+\cdots +\beta_{n}x_{i,N}$$

where *x*_*i*_ denotes the i'th data point, *y*_*i*_ its respective class, *x*_*ij*_ the j'th feature in *x*_*i*_, *β*_*i*_ a parameter and *N* the total number of features. The probability of *y*_*i*_ = 1 can be calculated by taking the inverse of the log-odds, which is the logistic function:

(2) $$P\left(y_{i}=1|x_{i}\right)=\frac{1}{1+e^{-\left(\beta_{0}+\beta_{1}x_{i,1}+\cdots +\beta_{n}x_{i,N}\right)}}$$

where *P*(*y*_*i*_ = 0|*x_i_*) is 1 − *P*(*y*_*i*_ = 1|*x_i_*) and the class with the highest probability is taken as its final prediction.

Generally, the algorithm is optimized using a gradient descent algorithm that minimizes the error between the predicted class value and its true class value by estimating the parameters *β*.

LR has been shown to be effective on task classification tasks in previous research (Komarek and Moore, 2003; Indra et al., 2016; Pranckevičius and Marcinkevičius, 2017).

#### 2.1.2 Support vector machine (SVM)

SVM is an algorithm that aims to find the most optimal hyperplane that separates data points from one class from the data points from another class (Boser et al., 1992; Cortes and Vapnik, 1995; Noble, 2006). The most optimal hyperplane is defined as the hyperplane with the largest margin between the classes, i.e. the distance between the plane and the closest data point of all classes is maximized. These closest points are called the support vectors and they completely determine the hyperplane. SVMs use kernel functions (Schölkopf et al., 2018) to be able to transform the data into a higher dimensional space such that the data is linearly separable, even when it would not be linearly separable in the original dimension of the data. The optimization problem that needs to be solved in an SVM is to calculate the maximum distance from the support vectors to the hyperplane, which can be computed through Langrange multipliers, and is expressed in the following equation:

(3) $$\max_{\alpha_{i}}\overset{N}{\underset{i=1}{\sum }}\alpha_{i}-\overset{N}{\underset{i=1}{\sum }}\overset{N}{\underset{j=1}{\sum }}\alpha_{i}\alpha_{j}y_{i}y_{j}k\left(x_{i},x_{j}\right)$$

where *x*_*i*_ and *x*_*j*_ are data points, *y*_*i*_ and *y*_*j*_ are their respective classes, *k*() is any kernel function, *N* is the total number of data points and *α*_*i*_ and *α*_*j*_ are the coefficients to be maximized for which holds *α*_*i*_ ≤ 0 and $\sum_{i=1}^{n}\alpha_{i}y_{i}=0$.

With the maximized values of *α*, the class of a binary problem can be calculated via:

(4) $$y_{i}=sign\left(b+\overset{N}{\underset{j=1}{\sum }}\alpha_{j}y_{j}k\left(x_{j},x_{i}\right)\right)$$

where *b* is given by:

(5) $$b=\frac{\sum_{i=1}^{N}y_{i}-\sum_{i=j}^{N}\alpha_{j}y_{j}k\left(x_{j},x_{i}\right)}{N}$$

SVMs are historically one of the most successful text classification algorithms and often outperform most other algorithms when it comes to text classification (Yang and Liu, 1999; Zhang and Oles, 2001; Mohammad et al., 2016).

#### 2.1.3 Naive Bayes (NB)

NB is an algorithm in which the probability that a data point belongs to a specific class is computed through Bayes' theorem, with the assumption that all features in the data point are independent of each other (Hand and Yu, 2001; Rish, 2001; Zhang, 2004). Bayes’ theorem is defined as follows:

(6) $$P\left(y_{i}|x_{i}\right)=\frac{P\left(x_{i}|y_{i}\right)P\left(y_{i}\right)}{P\left(x_{i}\right)}$$

where *x*_*i*_ represents a data point and *y*_*i*_ represents its class.

Often, *P*(*x*_*i*_) is difficult to determine. Fortunately, it is a constant given the data and can therefore be omitted. With the features in *x*_*i*_ assumed to be independent and the denominator omitted, the probability of a class can be calculated by estimating:

(7) $$P\left(y_{i}|x_{i}\right)\propto P\left(y_{i}\right)\overset{M}{\underset{j=1}{\prod }}P\left(x_{i,j}|y_{i}\right)$$

where *x*_*i*,*j*_ is a feature from *x*_*i*_ and *M* is the total number of features in *x*_*i*_. *P*(*Y*) and *P*(*x*_*i*,*j*_|*Y*) are estimated directly from the data. As a last step the probabilities over the classes are normalized such that they sum to one and the class with the biggest probability is taken as its final prediction.

NB is often used in text classification as it is a fast and efficient algorithm, and has proven to be effective for classifying text (Colas and Brazdil, 2006; Ting et al., 2011; Pratama and Sarno, 2015).

#### 2.1.4 Random forest (RF)

RF is an algorithm that builds multiple binary decision trees in parallel to create an ensemble of decision trees to make a prediction (Ho, 1995; Breiman, 2001; Cutler et al., 2012). At each iteration of the algorithm a new tree is made, which is done in three steps. The first step is to select a random subset of the data with replacement, this to ensure each tree in the ensemble will be different and combat overfitting. Then a random number of features from the total set of features will be selected. As a third step the feature and threshold with the most error reduction is chosen according to the weighted Gini impurity *I*_*wg*_, which is a metric to represent that a data point is classified incorrectly if the distribution of the split is followed:

(8) $$I_{WG}=\overset{B}{\underset{i=1}{\sum }}\frac{N_{i}}{N}\left(1-\underset{\text{c}\in C}{\sum }P{\left(C=c|N_{i}\right)}^{2}\right)$$

where *B* is the number of branches, *N* is the number of data points distributed across the branches, *N*_*i*_ is the number of data points in branch *i* and *C* are the possible classes. Steps two and three will then be repeated until a branch only contains data points of one class. After all iterations have finished, the final prediction for each data point is made by taking a majority vote over all created decision trees. A single decision tree makes a prediction by following the path of the decision tree according to the given data point until it reaches an end node corresponding to a class.

RFs have been shown to be an effective algorithm in the domain of text classification in the last decade (Xu et al., 2012; Parmar et al., 2014; Onan et al., 2016).

#### 2.1.5 AdaBoost (AB)

AB is an algorithm that uses an ensemble of one-deep binary decision trees that are sequentially generated and learn from previous mistakes by assigning larger weights to the data points it classified incorrectly (Freund and Schapire, 1996; Schapire, 2013). At each iteration t the decision tree, representing only one feature, that has the lowest weighted error is selected. The error is calculated via:

(9) $$\varepsilon_{t}=\overset{N}{\underset{i=1}{\sum }}w_{i,t}\left[y_{i}\neq h\left(x_{i}\right)\right]$$

where *x*_*i*_ is a data point, *y*_*i*_ is its label, *N* is the total number of data points, *w*_*i*,*t*_ is the weight associated with data point *x*_*i*_ at time *t* and *h*() is the decision tree. Next, the weight of each data point is updated before the next iteration is executed. The weight of each data point starts at t = 1 with 1/N and is each iteration updated according to:

(10) $$w_{i,t+1}=\frac{w_{i,t}}{Z}e^{-y_{i}\alpha_{t}h_{t}\left(x_{i}\right)}$$

where *Z* is a normalization factor and *α*_*t*_ is defined as $\frac{1}{2}ln\frac{1-\varepsilon_{t}}{\varepsilon_{t}}$. The amount of iterations is defined as a parameter. The final prediction is a weighted majority vote over all decision trees that are weighted according to their corresponding alpha value.

AB has been applied successfully on text classification tasks in previous research (Schapire et al., 1998; Bloehdorn and Hotho, 2004; Zhang et al., 2016).

#### 2.1.6 Gradient boosting (GB)

GB is very similar to AB and also sequentially generates one-deep binary decision trees to make an ensemble of trees. However, GB does not update the weight of data points in order to steer the decision trees in the right direction, but it uses gradient descent instead (Mason et al., 2000; Friedman, 2001; Ruder, 2016). The goal is to improve the predictions sequentially by minimizing a (differentiable) loss function using gradient descent by fitting each next decision tree on the residual error of the previous decision tree. The residual error at iteration *t* is calculated for each *i* ∈ {1, 2, …, *N*} as follows:

(11) $$\varepsilon_{i,t}=-\left[\frac{\partial l\left(y_{i},m_{t-1}\left(x_{i}\right)\right)}{\partial m_{t-1}\left(x_{i}\right)}\right]$$

where *N* is the total number of data points, *l*() represents the loss function, *x*_*i*_ is a data point, *y*_*i*_ is its label and *m*() is the incremental model defined as:

(12) $$m_{t}\left(x_{i}\right)=m_{t-1}\left(x_{i}\right)+\gamma h_{t}\left(x_{i}\right)$$

with *γ* the learning rate and *h*(*x*_*i*_) is the decision tree that minimizes the residual error. The number of iterations is set as a parameter. The final prediction is the output of the model in the last iteration.

GB has been proven as a successful text classification algorithm in the last few years (Prasad et al., 2017; Ramraj et al., 2018; Alzamzami et al., 2020).

#### 2.1.7 Long short-term memory (LSTM)

LSTM is a type of neural network that is capable of learning long-term dependencies in the input while processing it sequentially from left to right (Hochreiter and Schmidhuber, 1997; Gers et al., 1999; Greff et al., 2016), which is especially useful when looking at text. These long-term dependencies are learned by keeping a memory of the input that was seen before. This memory is used as a second input in each layer of the neural network next to the standard sequential input, and is produced by the previous layer. The memory output of an LSTM layer at step *t* is given by:

(13) $$c_{t}=f_{t}*c_{t-1}+i_{t}*{\tilde{c}}_{t}$$

where the * operator denotes element-wise multiplication and *f*_*t*_, *i*_*t*_ and *c* are given by:

(14) $$f_{t}=sigmoid\left(W_{f}x_{i,t}+W_{f}h_{t-1}+b_{f}\right)$$

(15) $$i_{t}=sigmoid\left(W_{i}x_{i,t}+W_{i}h_{t-1}+b_{i}\right)$$

(16) $$\tilde{c}=tanh\left(W_{c}x_{i,t}+W_{c}h_{t-1}+b_{c}\right)$$

with *x*_*i*,*t*_ the input of the model from data point *x*_*i*_ at time-step *t*, *W* the learned weight matrices and *b* the learned bias vectors. Furthermore, the output vector *h* at time step *t*, which together with the memory output will be the input for the next LSTM layer, is given by:

(17) $$h_{t}=sigmoid\left(W_{o}x_{t}+W_{o}h_{t-1}+b_{o}\right)*tanh\left(c_{t}\right)$$

The weight matrices and bias vectors are learned during training via a gradient descent algorithm. The model can make a prediction by feeding the output of the LSTM layers to one or more so called fully connected layers, expressed by:

(18) y=σ(Wa+b)

with *y* the output class, *a* the input to the layer and where *σ* can be any activation function, like a sigmoid or tanh. The last fully connected layer will output a probability for the model for each of the classes using a sigmoid function, where the final prediction is the class with the highest probability.

With the rise of neural networks, LSTMs have become a popular and successful method for text classification (Khanpour et al., 2016; Nowak et al., 2017; Mascio et al., 2020).

#### 2.1.8 Bidirectional encoder representations from transformers (BERT)

BERT is a neural network that can learn context in a sentence both from left to right and from right to left by processing all words from a sentence at the same time (Devlin et al., 2018; Jawahar et al., 2019). BERT consists of blocks called encoders. The amount of encoder blocks is a parameter of the algorithm. An encoder consists of an attention layer and two fully connected layers (see equation (18)). An attention layer calculates for each word in a sentence its relevance with the other words in the first encoder block, and in later blocks the relevancy for each element in the output vectors of the previous encoder. The attention layer makes use of so called multi-head attention, meaning that the relevancy is calculated multiple times using different learned weights, to simulate different perspectives on the relevancy between words. An attention layer is defined as follows:

(19) $$a_{i}=\left(h_{i,1},h_{i,2},\ldots ,h_{i,M}\right)W$$

with *i* ∈ {1, 2, …, *N*}, *N* the number of words in the sentence, *h* an attention head, *M* the number of chosen attention heads and *W* a learned weight matrix. The attention heads *h* are given by:

(20) $$h_{i,m}=\overset{N}{\underset{j=1}{\sum }}softmax_{j}\left(\frac{Q_{m}x_{i}K_{m}x_{j}}{Z}\right)V_{m}x_{j}$$

where *x*_*i*_ denotes the i'th word in the sentence, *Q*, *K* and *V* denote learned weight matrices and *Z* is a normalization factor. All weight matrices are learned during training via a gradient descent algorithm.

The final prediction is made by an added fully connected layer on top of the model with a sigmoid function to produce a probability for each of the classes and selecting the one with the highest probability per sentence. BERT has an advantage over other models, because it is pre-trained on the entire English Wikipedia^5^ and BookCorpus (Zhu et al., 2015) texts. This means that it has already captured a large amount of text representations before it is even trained on the task at hand and will therefore perform better at language understanding.

BERT is one of the newest advances in natural language modeling and is state-of-the-art in various text data sets (Sun et al., 2019; Aggarwal et al., 2020; González-Carvajal and Garrido-Merchán, 2020).

#### 2.1.9 Ensemble models

Since previous research has shown a better performance of ensembles of models compared to individual models, ensemble models were also investigated in this study. To this end, all unique ensemble combinations with at least two models (i.e. 247 combinations) were tested. The final classification by the ensembles was determined by summing the predicted probabilities of all involved trained models and averaging them.

### 2.2 Data collection

This research builds upon the data collected in two systematic reviews performed for the NVWA to make an inventory of chemical hazards in the supply chain of cereals and leafy greens (Kluche et al., 2020; Banach et al., 2019). An overview of their data collection procedure will be presented here. The literature for the systematic reviews was collected from Scopus and Web of Science using search queries defined by experts (see Appendix B). Collected articles were subsequently screened by an expert based on their title and abstract and categorized as either i) relevant, ii) maybe relevant or iii) not relevant. A second expert validated the decisions of the first expert by screening 10% of the collected articles independently. Inconsistencies were discussed and, if necessary, updated in the final evaluation. The evaluation was recorded in an Endnote^6^ file, containing the metadata from each article, including elements like the title, abstract and authors. Only English texts were considered relevant during the screenings. The first systematic review focused on the chemical contaminants found in the food chain of cereals such as wheat, oat, corn, rice and barley (Kluche et al., 2020). Only raw materials were taken into account and not processed cereal products, like bread or cornflakes. For the systematic review literature from the years 2008–2018 was used. In total 775 articles were screened. This resulted in 297 articles deemed to be relevant, 387 articles deemed to be not relevant and 91 articles were considered maybe relevant. The second systematic review focused on chemical contaminants in the food chain of leafy greens (Banach et al., 2019). Vegetables like lettuce, cabbage, spinach, kale and arugula were evaluated. Literature from the years 2009–2019 was used for the systematic review. In total 421 articles were screened. Of those articles, 70 articles were deemed to be relevant, 165 articles were deemed to be not relevant and 186 articles were considered maybe relevant.

To test whether the learned models used in this study are generalizable to new data from future years which can contain topics not covered in the current data, the same experts who performed the systematic reviews updated the systematic review with literature up until February 2020. The new found literature and their relevance category were put in a new data set, from now on called the future set. In order to be able to compare future data over the same number of years for the two topics, it was decided to move all the literature from the original leafy greens systematic review from 2019 to the future set so that both future sets contained data from 2019 up until February 2020. This meant moving four relevant articles, five not relevant articles and seven maybe relevant articles to the leafy greens future set.

Due to the ambiguous value of the articles that were categorized as maybe relevant, it was decided to not take them into account for this study to prevent training the machine learning algorithms on inconsistent data. The articles are classified as such because they either describe field studies in countries not relevant for the Dutch food safety market or if there is a possibility useful information is mentioned about chemical hazards in the body text even though the article is not on the topic of identification of chemical hazards. These articles can be looked through by the experts to possibly find more information if for a certain hazard group not a satisfactory number of articles were found within the relevant articles, but they are often not found relevant.

This results in final data sets of 684 articles for the cereals case of which 297 were considered relevant (43.3%), and 226 articles for the leafy greens case of which 66 were deemed relevant (29.2%). The future set consists of 147 articles for the topic of cereals with 71 relevant articles (48.2%) and 96 articles for the topic of leafy greens with 62 relevant articles (64.6%). All articles were exported from Endnote to a BibTeX file to make the data machine-readable. From this file only the titles and abstracts were collected. The title and abstract were concatenated per article to form one data entry and the entry was labelled as either relevant or not relevant.

### 2.3 Data preprocessing

Preprocessing of the data is a necessary step as the algorithms need numerical instead of textual input. The LSTM and BERT algorithms were given a different preprocessing approach to the rest of the algorithms as they are capable of handling sequential data. The other six algorithms handle text data as bag-of-words representations, in which word order is ignored and only the unique words are kept. First, all words are converted into lower-case and all symbols, numbers and stop words are removed. Stop words are words that carry no real semantic meaning (e.g. articles and prepositions) and can be removed in order to focus on the words that represent the subject of a text and prevent uninformative features. Stemming of the words was also tested as preprocessing step, but this did not improve performance. Next, the number of unique words in the text the algorithms are trained on determines the length of the feature vector. Each input text is represented by this feature vector filled with a TF-IDF feature for each unique word (Robertson and Jones, 1976). TF-IDF features are one of the most popular features for text and represents the importance of a word in the entire document. In contrast to the frequency of a word, TF-IDF is normalized by the number of data points that contain the word to penalize more common words.

In the preprocessing for the LSTM and BERT, the specific order of the words is kept and no stop words are deleted. All words get transformed to lower case and all symbols and numbers are removed. Words are then transformed into numerical vectors where each unique words gets a unique number. Neural networks require each input to have the same length to be able to do the computations, so each data point is padded at the end of the vector with padding tokens to the longest text in the data the algorithms are trained on. These padding tokens are ignored during learning, so do not influence the performance of the model.

Data augmentation was implemented as an extra preprocessing step to combat the imbalance between the amount of relevant and not relevant articles and increase the total amount of data available. The two cases used in this study only contained a few hundred data points and, in addition, the leafy greens case is quite imbalanced with only 29% of data in the relevant class. Two data augmentation techniques were implemented and set as optional parameters for each algorithm: Synthetic minority over-sampling technique (SMOTE) (Chawla et al., 2002) and general synthetic over-sampling (SO).

SMOTE generates new data points for the minority class by selecting a random data point in that class and updating the values in the feature vector so that they lie in between the original values and the values of one of the three nearest neighbors selected by the KNN algorithm (Fix and Hodges, 1951). The number of extra data points that is created via SMOTE is equal to the difference in data points between the minority and majority class. SO generates new data points for all classes independent of their imbalance. The same technique behind SMOTE was used to create new data points. Twenty percent of the data points in the training set were used to generate new data points leading to a new training data set of 120% the original size. Note that when SMOTE and SO are used together, SMOTE will be applied first.

### 2.4 Training and validation

The two data sets for cereals and leafy greens (excluding the future sets) were randomly split into a training set and a test set. The training set consisted of 80% of the data and the test set consisted of the remaining 20%. The training set was trained using 5-fold cross-validation, where the data is split into five different parts. Each algorithm is trained five times, each training round the algorithm uses four parts of the data as training data and one part as validation data. The average validation performance over the five training rounds was seen as the final validation performance. Performance was measured using three metrics: precision, recall and F1 score (Goldstein et al., 1999; Sokolova et al., 2006). Precision represents the probability that a data point is classified correctly as its class out of all data point classified as that class. Recall on the other hand represents the probability that a data point is classified correctly out of all the data points that actually belong to that class. Mathematically, precision (*pr*) and recall (re) of a class *c* is expressed as follows:

(21) $$pr\left(c\right)=\frac{TP_{c}}{TP_{c}+FP_{c}}$$

(22) $$re\left(c\right)=\frac{TP_{c}}{TP_{c}+FN_{c}}$$

where *TP*_*c*_ is the number of correctly classified data points in class *c*, *FP*_*c*_ is the number of data points that is incorrectly classified as class *c* and *FN*_*c*_ is the data points that are not classified as class *c* but should have been.

F1 score combines precision and recall is a single metric and is calculated as the harmonic mean of precision and recall:

(23) $$F1\left(c\right)=2\frac{pr\left(c\right)re\left(c\right)}{pr\left(c\right)+re\left(c\right)}$$

Classifications of the data points were determined by thresholding the predicted probabilities by 0.5. All probabilities above and equal to 0.5 were classified as relevant and probabilities below 0.5 were classified as not relevant.

The final training parameters for the algorithms were determined based on the combination of parameters that achieved the highest average validation performance across the two different cases. Performance was based on the average F1 score of the relevant and not relevant class. The two data augmentation techniques, however, were selected per case to account for the different imbalances and number of data points. Each algorithm was trained using the final parameter set on the entire training data to create the final model.

The final parameters of each algorithm are described below. LR was trained using 5 iterations and L2 regularization (Wahba, 1995) with a regularization factor of 0.001. The SVM was trained using a linear kernel and L2 regularization with a regularization factor of 1.0. NB used an alpha value of 1.0. RF used 1000 decision trees and considered at each split a random amount of features equal to the square root of the total number of features. AB also used 1000 decision trees. GB used 2000 decision trees, a learning rate of 0.01 and Friedman mean squared error as the loss function. The LSTM consisted of four layers: An embedding layer, a bidirectional LSTM layer and two fully connected layers. The LSTM layer consisted of 12 nodes and the fully connected layers of 12 nodes and 1 node respectively. In between each layer dropout was applied with a rate of 0.5. It was trained for 50 epochs with a learning rate of 0.0005 and a L2 regularization factor of 0.0001. The batch size was 32 and during training each batch was balanced across the two classes. BERT was initialized with the DistilBERT parameters (Sanh et al., 2019), which is a smaller pretrained BERT model more suitable for small data sets, consisting of 6 encoder blocks and 12 attention heads. The attention layer contains 768 nodes, the fully connected layers in the encoders contain 3072 nodes and the final fully connected layer contains 2 nodes. Dropout was applied after each layer and with a rate of 0.1. It was trained for 3 epochs using a learning rate of 5^−5^ and a one-cycle policy (Smith, 2018). The batch size was 2, due to the large GPU memory requirement of the network.

The parameters for the data augmentation can be found in Table 1, and are represented by a Boolean value. True indicates that type of augmentation was applied for that combination of algorithm and data set in the final model and False means that it was not applied. In the cereals case, data augmentation did not lead to improved performance for any of the algorithms. For the leafy greens case SMOTE improved performance for six out of the eight algorithms, while SO improved performance for three algorithms. Note that SO only proved beneficial in sequence with SMOTE and never on its own.

**Table 1:** The data augmentation parameters for each of the algorithms in the two data cases: cereals and leafy greens.

Algorithm	Cereals		Leafy greens	
	SMOTE	SO	SMOTE	SO
AdaBoost	False	False	True	True
BERT	False	False	True	False
Gradient boosting	False	False	True	True
Logistic Regression	False	False	True	False
LSTM	False	False	False	False
Naive Bayes	False	False	True	True
Random forest	False	False	True	False
Support vector machine	False	False	False	False

## 3 Results

The performance of the trained models on the test set and the future set can be found in Table 2 and Table 3 for the cereals and leafy greens cases, respectively. Precision, recall and F1 score are shown for the relevant class, the not relevant class and the average across the two classes. The best values per column for the two sets are indicated in bold.

**Table 2:** Performance of the trained models on the test and future set from the systematic review on cereals. Performance is shown in terms of precision, recall and F1 score for the relevant and not relevant class. An average across the two classes is also shown. The best values per column and set are boldfaced.

Algorithm	Set	Relevant			Not relevant			Average		
		pr	re	F1	pr	re	F1	pr	re	F1
AB	Test set	75.0%	76.4%	75.7%	84.0%	82.9%	83.4%	79.5%	79.6%	79.6%
	Future set	79.4%	70.4%	74.6%	75.0%	82.9%	78.7%	77.2%	76.7%	76.7%
BERT	Test set	80.0%	87.3%	83.5%	90.9%	85.4%	88.1%	85.5%	86.3%	85.8%
	Future set	**91.1%**	71.8%	80.3%	78.0%	**93.4%**	85.0%	84.5%	82.6%	82.7%
GB	Test set	81.2%	70.9%	75.7%	82.0%	89.0%	85.4%	81.6%	80.0%	80.6%
	Future set	85.5%	66.2%	74.6%	73.9%	89.5%	81.0%	79.7%	77.8%	77.8%
LR	Test set	**83.9%**	85.5%	**84.7%**	90.1%	**89.0%**	**89.6%**	**87.0%**	**87.2%**	**87.1%**
	Future set	90.0%	76.1%	82.4%	80.5%	92.1%	85.9%	85.2%	84.1%	84.2%
LSTM	Test set	80.4%	67.3%	73.3%	80.2%	89.0%	84.4%	80.3%	78.1%	78.8%
	Future set	90.6%	67.6%	77.4%	75.5%	93.4%	83.5%	83.0%	80.5%	80.5%
NB	Test set	76.9%	**90.9%**	83.3%	**93.1%**	81.7%	87.0%	85.0%	86.3%	85.2%
	Future set	85.7%	84.5%	85.1%	85.7%	86.8%	86.3%	85.7%	85.7%	85.7%
RF	Test set	75.4%	78.2%	76.8%	85.0%	82.9%	84.0%	80.2%	80.6%	80.4%
	Future set	87.3%	77.5%	82.1%	81.0%	89.5%	85.0%	84.1%	83.5%	83.5%
SVM	Test set	81.4%	87.3%	84.2%	91.0%	86.6%	88.8%	86.2%	86.9%	86.5%
	Future set	91.0%	**85.9%**	**88.4%**	**87.5%**	92.1%	**89.7%**	**89.3%**	**89.0%**	**89.1%**

**Table 3:** Performance of the trained models on the test and future set from the systematic review on leafy greens. Performance is shown in terms of precision, recall and F1 score for the relevant and not relevant class. An average across the two classes is also shown. The best values per column and set are boldfaced.

Algorithm	Set	Relevant			Not relevant			Average		
		pr	re	F1	pr	re	F1	pr	re	F1
AB	Test set	80.0%	57.1%	66.7%	83.3%	93.8%	88.2%	81.7%	75.4%	77.5%
	Future set	88.9%	64.5%	74.8%	56.9%	85.3%	68.2%	72.9%	74.9%	71.5%
BERT	Test set	70.6%	**85.7%**	77.4%	**93.1%**	84.4%	88.5%	81.8%	**85.0%**	83.0%
	Future set	85.9%	88.7%	87.3%	78.1%	73.5%	75.8%	82.0%	81.1%	81.5%
GB	Test set	81.8%	64.3%	72.0%	85.7%	93.8%	89.6%	83.8%	79.0%	80.8%
	Future set	85.4%	56.5%	68.0%	50.9%	82.4%	62.9%	68.1%	69.4%	65.4%
LR	Test set	76.9%	71.4%	74.1%	87.9%	90.6%	89.2%	82.4%	81.0%	81.7%
	Future set	87.8%	69.4%	77.5%	59.6%	82.4%	69.1%	73.7%	75.9%	73.3%
LSTM	Test set	62.5%	71.4%	66.7%	86.7%	81.2%	83.9%	74.6%	76.3%	75.3%
	Future set	83.9%	83.9%	83.9%	70.6%	70.6%	70.6%	77.2%	77.2%	77.2%
NB	Test set	64.7%	78.6%	71.0%	89.7%	81.2%	85.2%	77.2%	79.9%	78.1%
	Future set	84.5%	**96.8%**	**90.2%**	**92.0%**	67.6%	**78.0%**	**88.3%**	**82.2%**	**84.1%**
RF	Test set	**81.8%**	64.3%	72.0%	85.7%	**93.8%**	89.6%	83.8%	79.0%	80.8%
	Future set	88.1%	59.7%	71.2%	53.7%	85.3%	65.9%	70.9%	72.5%	68.5%
SVM	Test set	78.6%	78.6%	**78.6%**	90.6%	90.6%	**90.6%**	**84.6%**	84.6%	**84.6%**
	Future set	**90.0%**	72.6%	80.4%	63.0%	**85.3%**	72.5%	76.5%	78.9%	76.4%

For the cereals case in Table 2, LR was the best performing model based on the test set. It acquired the best score for seven out of the nine columns and has the best F1 score for both the relevant and not relevant classes. However, for the future set the SVM performed best. It also obtained the best score for seven out of nine columns and has the best average F1 score. For the leafy greens case in Table 3, the SVM performed best on the test set. With four out of nine columns containing the highest score and the best F1 score across the two classes, it achieved the best scores among the models. For the future set, the NB model performed best with the highest scores in seven out of nine columns and the best F1 scores in both classes. Considering the performance across the two cases over the two sets, the model with the highest average F1 score was the SVM with a score of 84.2% followed by NB with a score of 83.3% and BERT with a score of 83.2%.

In addition to these eight individual models, ensemble models were created to test if a combination of models could lead to a better performance. In total 247 combinations (representing all unique combinations with at least two models) were made and tested on the test and future set for both the cereals and leafy green case. The results of the ensemble models can be found in Table 4 and Table 5 for the cereals and leafy greens cases, respectively. Only the top five best ensemble models are presented per combination of each case and set.

**Table 4:** Performance of the top five best ensemble models on the test and future set from the systematic review on cereals. Performance is shown in terms of precision, recall and F1 score for the relevant and not relevant class. An average across the two classes is also shown.

Ensemble top 5	Set	Relevant			Not relevant			Average		
		pr	re	F1	pr	re	F1	pr	re	F1
1. NB, SVM	Test set	82.0%	90.9%	86.2%	93.4%	86.6%	89.9%	87.7%	87.7%	88.0%
2. AB, LR, NB, RF, SVM	Test set	83.1%	89.1%	86.0%	92.3%	87.8%	90.0%	87.7%	88.4%	88.0%
3. GB, NB, SVM	Test set	84.2%	87.3%	85.7%	91.2%	89.0%	90.1%	87.7%	88.1%	87.9%
4. GB, LR, NB, SVM	Test set	84.2%	87.3%	85.7%	91.2%	89.0%	90.1%	87.7%	88.1%	87.9%
5. AB, GB, NB, SVM	Test set	84.2%	87.3%	85.7%	91.2%	89.0%	90.1%	87.7%	88.1%	87.9%
1. NB, SVM	Future set	91.3%	88.7%	90.0%	89.7%	92.1%	90.9%	90.5%	90.4%	90.5%
2. AB, NB, SVM	Future set	91.3%	88.7%	90.0%	89.7%	92.1%	90.9%	90.5%	90.4%	90.5%
3. AB, SVM, NB	Future set	91.0%	85.9%	88.4%	87.5%	92.1%	89.7%	89.3%	89.0%	89.1%
4. RF, SVM, AB	Future set	91.0%	85.9%	88.4%	89.3%	89.0%	89.7%	89.3%	89.0%	89.1%
5. NB, RF, SVM	Future set	91.0%	85.9%	88.4%	87.5%	92.1%	89.7%	89.3%	89.0%	89.1%

**Table 5:** Performance of the top five best ensemble models on the test and future set from the systematic review on leafy greens. Performance is shown in terms of precision, recall and F1 score for the relevant and not relevant class. An average across the two classes is also shown.

Ensemble top 5	Set	Relevant			Not relevant			Average		
		pr	re	F1	pr	re	F1	pr	re	F1
1. AB, SVM	Test set	78.6%	78.6%	78.6%	90.6%	90.6%	90.6%	84.6%	84.6%	84.6%
2. BERT, SVM	Test set	78.6%	78.6%	78.6%	90.6%	90.6%	90.6%	84.6%	84.6%	84.6%
3. NB, SVM	Test set	78.6%	78.6%	78.6%	90.6%	90.6%	90.6%	84.6%	84.6%	84.6%
4. AB, BERT, SVM	Test set	78.6%	78.6%	78.6%	90.6%	90.6%	90.6%	84.6%	84.6%	84.6%
5. AB, NB, SVM	Test set	78.6%	78.6%	78.6%	90.6%	90.6%	90.6%	84.6%	84.6%	84.6%
1. AB, NB	Future set	84.3%	95.2%	89.4%	88.5%	67.6%	76.7%	86.4%	81.4%	83.0%
2. LR, NB, SVM	Future set	91.1%	82.3%	86.4%	72.5%	85.3%	78.4%	81.8%	83.8%	82.4%
3. BERT, NB	Future set	85.1%	91.9%	88.4%	82.8%	70.6%	76.2%	83.9%	81.3%	82.3%
4. AB, BERT, NB	Future set	85.1%	91.9%	88.4%	82.8%	70.6%	76.2%	83.9%	81.3%	82.3%
5. NB, SVM	Future set	89.7%	83.9%	86.7%	73.7%	82.4%	77.8%	81.7%	83.1%	82.2%

For the cereal case presented in Table 4 an ensemble of NB and SVM achieved the best results for both the test and future set. For the leafy greens case presented in Table 5, the top five ensembles for the test set all achieved the same score, e.g. combining either AB, BERT or NB with SVM all yield the top score. On the future set an ensemble of AB and NB performed best. Considering the ensembles across the two cases over the two sets, there was only one ensemble that occurred in all four top five's: an ensemble of NB and SVM. This ensemble achieved the best score in both sets of the cereals case and in the test set of the leafy greens case. All three scores are higher than the respective best scores achieved by the single models. In the future set of the leafy greens case it achieved the fifth best score with a difference in score of 0.8% with the best score in that set and it had a difference of 1.9% with the respective best score achieved by the single models. The average F1 score of the NB and SVM ensemble across the two cases over the two sets was 86.3%, which was the highest average across all individual models and ensemble models. The corresponding averages for precision and recall are 85.4% and 85.5% for the relevant class and 86.9% and 87.9% for the not relevant class. This model results in an average decrease of 54.4% in the amount of articles the reviewer has to read and an average decrease in irrelevant articles of 87.9% across the cereals and leafy greens cases over the test set and future set.

However, a successful model should have a high recall for the relevant class to ensure that a significant number of relevant articles will not be omitted from the final selection. The current result of the NB and SVM ensemble with a relevant recall of 85.5% means that 14.5% of the relevant articles will not be included in the final selection and therefore will not be seen by the reviewer. This can be remedied by lowering the probability threshold, which will make sure articles are classified as relevant more quickly. This will increase the recall, but also decrease the precision for the relevant class. A recall of at least 95% was desired to warrant that a significant number of relevant articles will not be lost, while not being overly accepting, which would negatively affect the performance of the model. The first threshold to cross an average recall of 95% in the relevant class over the data sets was a threshold of 0.25, which lead to an average recall of 96.5% and an average precision of 65.0% in the relevant class and an average recall of 57.8% and an average precision of 96.7% in the not relevant class (see Table 6). Applying this threshold results in an average decrease of 32.8% in the amount of articles the reviewer has to read and an average decrease in irrelevant articles of 57.8% across the cereals and leafy greens cases over the test set and future set.

**Table 6:** Performance of the best ensemble model (NB and SVM) with a threshold of 0.25 on the test and future set from the systematic review on cereals and leafy greens. Performance is shown in terms of precision, recall and F1 score for the relevant and not relevant class. An average across the two classes and an average across the data sets is also shown.

Case	Set	Relevant			Not relevant			Average		
		pr	re	F1	pr	re	F1	pr	re	F1
Cereals	Test set	57.1%	94.5%	71.2%	93.5%	52.4%	67.2%	75.3%	73.5%	69.2%
	Future set	71.4%	98.6%	82.8%	98.0%	63.2%	76.8%	84.7%	80.9%	79.8%
Leafy greens	Test set	52.0%	92.9%	66.7%	95.2%	62.5%	75.5%	73.6%	77.7%	71.1%
	Future set	79.5%	100.0%	88.6%	100.0%	52.9%	69.2%	89.7%	76.5%	78.9%
**Average**		**65.0%**	**96.5%**	**77.3%**	**96.7%**	**57.8%**	**72.2%**	**80.8%**	**77.2%**	**74.8%**

## 4 Discussion

Eight different machine learning algorithms (LR, NB, SVM, RF, AB, GB, LSTM and BERT) were implemented and trained on the data of the screening stage of two different systematic review cases: chemical hazards in cereals and chemical hazard in leafy greens. The trained models and all possible unique ensemble combinations of these models were tested on a held-out set of the data for evaluation. It was shown that an ensemble of NB and SVM performed best across all single models and ensembles. Across the two cases and the two sets, the ensemble resulted in an average decrease of 32.8% in the amount of articles the reviewer has to read and an average decrease in irrelevant articles of 57.8% when adhered to a recall of 95%. The reduction of articles could even be increased if lower levels of recall are acceptable, but this can lead to a less complete systematic review as some relevant articles will be missed. Increasing the recall to 100% would also not be advisable as this would enforce the model to be overly accepting, resulting in a negative effect on the overall performance. Furthermore, since the class labels of the data were set by human reviewers, who can make mistakes in their labelling during systematic reviews (Wang et al., 2020), it is better to allow a bit of room in the recall of the model.

Even though the number of articles to be screened in a systematic review on the domain of food safety is relatively small, reducing the burden of screening with a machine learning model will still have a positive impact. The expert will have to spend less hours scanning through articles, which saves costs and lessens the monotonous part of writing a systematic review. Furthermore, since the process of data collection from literature databases like Scopus and Web of Science can be automated through their APIs, a system that collects and classifies new articles automatically can be set up. This way articles classified as relevant can be shown to the experts in real-time, so they can stay on top of the topic and make a more informed decision if a new systematic review is needed because of changes in the respective food supply chain.

The good performance of the ensembles compared to the single models shows the power of combining multiple models together. The SVM and NB were the two best single performing models, but still were able to complement each other to increase performance in the ensemble. The averaging across the probabilities ensured some mistakes made by one model to be corrected by the other. It must be noted that the selection of the specific ensemble is very important. Different ensembles performed well on each data set, the ensemble of the SVM and NB was the only ensemble present in all top five best ensembles across the different data sets. It is apparently not sufficient to just combine two or more well performing models to create an ensemble that performs better than the models separately. However, for a systematic review data set with only hundreds of articles an ensemble of an SVM and NB has proven to perform consistently well and would be a good choice.

Comparing the classifications of the individual models does show a trend in what articles are classified correctly and incorrectly. Articles that not discuss chemical contaminants, but instead discuss microbiological contaminants or quality of product, will almost always be classified as not relevant. This holds for literature describing the development of a novel detection method that could be used for chemical contaminants or the effect of the contaminants on human health as well. Contrarily, articles solely describing the concentration of chemical contaminants found in cereals and leafy greens will mostly be classified as relevant. It gets difficult when articles discuss chemical contaminants, but don't fall in the scope of the review. Examples of this are chemical contaminants in processed products, the effects of chemical contaminants on growth and yield, or risk management systems, which often are falsely classified as relevant. Reversely, articles discussing both microbial and chemical hazards or new detection methods that are applied in the field directly can be falsely classified as not relevant. These more difficult articles are the distinguishing factor between the performance of the models.

The success of the SVM both as a single model and combined in an ensemble is in line with previous work, where four out of the six studies were most successful with an SVM (Wallace et al., 2010; Bekhuis and Demner-Fushman, 2012; García Adeva et al., 2014; Timsina et al., 2016). SVMs have historically always performed well on text classification (Yang and Liu, 1999; Zhang and Oles, 2001; Mohammad et al., 2016), because of their ability to generalize well on a large number of features (Joachims, 1998; Leopold and Kindermann, 2002). However, they have since been surpassed by neural network models like LSTM and BERT as the state-of-the-art (Lee and Dernoncourt, 2016; Mascio et al., 2020; Hu et al., 2020). Nonetheless, neural networks often only perform optimally when there is a large data set. In the present study, the amount of data is limited, which would explain why the more traditional models like SVM and NB perform better.

The amount of data can also explain why the presented F1 scores are higher for the cereals case than for the leafy greens case as the training data had a size of 547 and 180 articles, respectively. Interestingly, this difference in F1 scores almost disappears when lowering the threshold to 0.25 for the ensemble of NB and SVM. This suggests that the models are less certain of their classification in the leafy greens case by attributing a lower probability to articles belonging to the relevant class, possibly because the models had less data to train on. The fact that at a lower threshold the leafy greens models achieve similar F1 scores to the cereals models indicates that even cases with a low amount of data can improve from automatic classification through machine learning.

One of the strengths of combining a machine learning model with a human reviewer lies in the fact that the model can keep improving with each use. After the model has made the initial selection of possible relevant articles from a new unseen data set, the human reviewer will produce a final ‘correct’ selection. This final selection of relevant and not relevant articles can be added to the training data of the model and increase the amount of data the model can train on. More data leads to improved performance and will decrease the amount of not relevant articles with the next use.

A limitation of the current work is that a model is trained per case, meaning that there needs to be training data available from that exact case from a previous systematic review. Moreover, the reviewer needs to have saved both the articles that were considered relevant and not relevant in order for the data to be useful. For new topics, the approach reported in this study is unfortunately not applicable out of the box. The reviewer will first need to spend some time labelling a good part of the data before a model can be trained and applied. However, there are tools available to aid a reviewer in screening the data in a way that not all data has to be seen with the use of machine learning, e.g. RobotAnalyst (Przybyła et al., 2018), SWIFT-Active (Howard et al., 2020) or ASReview (van de Schoot et al., 2021). These tools can reduce the time to label an article data set considerably by actively learning to identify relevant articles during the screening process and discarding the not relevant ones.

An additional limitation of this research is that the articles classified as maybe relevant were discarded from the training data. Ideally, all data should be incorporated in the training of the model as either relevant or not relevant to cover all possible input. Due to the ambiguous nature of the articles classified as maybe relevant, this was not possible currently, but in future it would be best to entangle the articles in this class and move them to either the relevant or not relevant set.

For future research, it could be investigated whether it is beneficial for performance to train a model on all available data independent of the case to create a model that detects general relevant food safety literature. It was observed that in the used data the context of the relevant cereals and leafy greens articles was very similar. Combining different cases together will lead to more data to train the model on, presumably leading to better performance, and could especially be beneficial for those cases that have little to no data available. Another approach that could be investigated for cases that have no previous data available is unsupervised learning, where labels are not required. Instead the articles would be clustered according to how similar they are in terms of words, topics or context.

In this study, only two systematic reviews could be included. Future work could apply the models to more systematic reviews covering a wider range of topics to investigate whether the results stay consistent. Furthermore, only the title and abstract were used, being the information the human reviewers base their assessment on. It is understandable that reading the entire article to determine its relevancy is infeasible for human reviewers, however, for a computer this poses less of a problem. Additional research can be done to explore the possibility of using (part of) the full article text as input for the classification models instead of using only the abstract. Access to full-text articles was historically quite limited, but with the push towards open science, more full-texts are steadily becoming available. Tools that convert PDF to text can be used to access the raw texts of the articles if those are not available. Research in extracting text from specifically PDFs of scientific articles has also been performed (Ramakrishnan et al., 2012; Tkaczyk et al., 2015; Yu et al., 2020). Challenges still exist when it comes to automatically parsing tabular and graphical content, but approaches have been developed to overcome these issues (Clark and Divvala, 2016; Singh et al., 2018; Siegel et al., 2018).

In order to save more time and automate more of the systematic review process, future work could also focus on also automatically collecting the relevant parts of the text from the selected relevant articles. For example by retrieving those paragraphs or sentences most likely to contain useful information by looking for certain sections and keywords. This would decrease time spent screening through the parts of the article that are not of importance to the review and present the reviewer with a better overview of the content.

## 5 Conclusion

In this study, the application of machine learning was demonstrated for the automatic classification of literature in systematic reviews on food safety. It was shown that the applied models are successful in the reduction of irrelevant articles, while retaining high percentages of relevant articles. Multiple machine learning algorithms and all possible ensemble combinations were tested and it was concluded that an ensemble of naive Bayes and a support vector machine performed best overall. By including a set with future literature, it was shown that the results do not only apply on the literature from the period the model trained on, but also on literature from the foreseeable future. The positive results show that human reviewers in a systematic review on food safety can benefit from using machine learning to do automatic classification of the literature, as it can save valuable time but does not comprise the completeness of the review.

## CRediT authorship contribution statement

**Leonieke M. van den Bulk:** Methodology, Software, Validation, Writing – original draft, Writing – review & editing. **Yamine Bouzembrak:** Conceptualization, Methodology, Writing – review & editing, Visualization, Supervision, Project administration. **Anand Gavai:** Methodology, Writing – review & editing. **Ningjing Liu:** Writing – review & editing. **Lukas J. van den Heuvel:** Writing – review & editing. **Hans J.P. Marvin:** Conceptualization, Methodology, Writing – review & editing, Supervision.

## Declaration of competing interest

The authors declare that they have no known competing financial interests or personal relationships that could have appeared to influence the work reported in this paper.

## Appendix A

The code and data used in this study can be found on https://github.com/WFSRBigData/systematic-review-classification.

## Appendix B

The search queries used by Kluche et al. (2020) and Banach et al. (2019) to collect the data for the systematic reviews can be found below. The data collection for the cereals case was done with two queries of which the results where combined together in one data set.

### Cereals:

#### Search-query 1:

In title: cereals or oat* or barley or rice or millet or rye or sorghum or wheat or maize or corn or poaceae or glycine or buckwheat or fonio or triticale.

AND

In title, abstract, keywords: “food contamination” OR “chemical pollutant*” OR “chemical hazard*” OR contamina* OR toxin* OR “toxic substance*” OR “toxic compound*” OR pollutant* OR “agricultural chemical*” OR “chemical compound*” OR “chemical substance*” OR residu*

AND

In title, abstract, keywords: “public health” OR “HACCP” OR “consumer protection” OR consumer* OR “food safety” OR “risk assessment*” OR “risk analys*” OR “hazard analys*” OR “human health*” OR “health impact” OR “health risk*”

AND NOT

In title, abstract, keywords: pathogen* OR streptococcus OR listeria OR virus OR bacillus OR salmonella OR clostridium OR staphylococcus OR outbreak OR “foodborne disease*” OR environment* OR ecological OR bioavailability OR “water management” OR soil OR nutritional*

AND NOT

In title: fung* OR method* OR experiment* OR analytic* OR model*

AND

Publication year: 2008–2018.

#### Search-query 2:

In title: cereals or oat* or barley or rice or millet or rye or sorghum or wheat or maize or corn or poaceae or glycine or buckwheat or fonio or triticale.

AND

In title, abstract, keywords: “food contamination” OR “chemical pollutant*” OR “chemical hazard*” OR contamina* OR toxin* OR “toxic substance*” OR “toxic compound*” OR pollutant* OR “agricultural chemical*” OR “chemical compound*” OR “chemical substance*” OR residu*

AND

In title, abstract, keywords: “public health” OR “HACCP” OR “consumer protection” OR consumer* OR “food safety” OR “risk assessment*” OR “risk analys*” OR “hazard analys*” OR “human health*” OR “health impact” OR “health risk*”

AND NOT

In title: pathogen* or streptococcus or listeria or virus or bacillus or salmonella or clostridium or staphylococcus or outbreak or “microb* contamin*” or “foodborne disease*” OR fung* or method* OR experiment* OR analytic* OR model* OR environment* or ecological.

AND

Publication year: 2008–2018.

AND

Document type: review.

### Leafy Greens:

#### Search-query:

In title: brocco* OR cauliflower* OR sprout* OR cabbage* OR chicory OR spinach* OR “turnip top*” OR “turnip green*” OR kale OR chard OR lettuce* OR endive OR escarole* OR “leafy vegetable*” OR “green vegetable*” OR “leafy vegetable*” OR salad OR choi OR choy OR artichoke OR arugula OR “beet green” OR bitterleaf OR celery OR celtuce OR “collard green*” OR *cress* OR epazote OR “garden rocket” OR komatsuna OR “mizuna greens” OR “mustard green*” OR “leaf mustard*” OR radicchio OR rapini OR tatsoi OR chaya OR chickweed OR “Chinese mallow” OR Chrysanthemum OR “fat hen” OR “fluted pumpkin” OR samphire OR “Greater plantain” OR “jute plant” OR karkalla OR “Lagos bologi” or orache OR purslane OR rucola OR sculpit OR stridolo OR soko OR “spleen amaranth”.

AND

In title, abstract or keywords: “food contamination” OR “chemical pollutant*” OR “chemical hazard*” OR contamina* OR toxin* OR “toxic substance*” OR “toxic compound*” OR pollutant* OR “agricultural chemical*” OR “chemical compound*” OR “chemical substance*” OR residu*

AND

In title, abstract or keywords: “public health” OR “HACCP” OR “consumer protection” OR consumer* OR “food safety” OR “risk assessment*” OR “risk analys*” OR “hazard analys*” OR “human health*” OR “health impact” OR “health risk*”

AND

In title: pathogen* OR streptococcus OR listeria OR *virus* OR bacillus OR salmonella OR clostridium OR staphylococcus OR outbreak OR “foodborne disease*” OR fung* OR campylobacter OR “*Escherichia coli*” OR “*E. coli*” OR model* OR analytic* OR microbio* OR bacteri* OR virol* Or nutri*

AND

Publication year: 2009–2019.

## Footnotes

1 Note that all recall and precision values discussed are those of the ‘relevant’ class.

2 https://www.scopus.com.

3 https://www.webofknowledge.com.

4 https://www.python.org.

5 https://en.wikipedia.org.

6 https://endnote.com.
